# Proinflammatory activity of VEGF-targeted treatment through reversal of tumor endothelial cell anergy

**DOI:** 10.1007/s10456-022-09863-4

**Published:** 2022-12-02

**Authors:** Patrycja Nowak-Sliwinska, Judy R. van Beijnum, Christian J. Griffioen, Zowi R. Huinen, Nadine Grima Sopesens, Ralph Schulz, Samir V. Jenkins, Ruud P. M. Dings, Floris H. Groenendijk, Elisabeth J. M. Huijbers, Victor L. J. L. Thijssen, Eric Jonasch, Florry A. Vyth-Dreese, Ekaterina S. Jordanova, Axel Bex, René Bernards, Tanja D. de Gruijl, Arjan W. Griffioen

**Affiliations:** 1 Angiogenesis Laboratory, Department of Medical Oncology, Amsterdam University Medical Center, Cancer Center Amsterdam, Vrije Universiteit Amsterdam, Cancer Center Amsterdam, Amsterdam, The Netherlands; 2 School of Pharmaceutical Sciences, Faculty of Sciences, University of Geneva, Patrycja Nowak-Sliwinska, Rue Michel-Servet 1, CMU, 1211 Geneva 4, Switzerland; 3 Institute of Pharmaceutical Sciences of Western Switzerland, University of Geneva, Geneva, Switzerland; 4 Department of Radiation Oncology, University of Arkansas for Medical Sciences, Little Rock, AR USA; 5 Division of Molecular Carcinogenesis, Oncode Institute, Amsterdam, The Netherlands; 6 Department of Genitourinary Oncology, MD Anderson Cancer Center, Houston, TX USA; 7 Division of Immunology, The Netherlands Cancer Institute, Amsterdam, The Netherlands; 8 Department of Urology, The Netherlands Cancer Institute, Amsterdam, The Netherlands; 9 Division of Surgery and Interventional Science, Royal Free London NHS Foundation Trust, University College London, Pond Street, London, UK; 10 Center for Gynaecologic Oncology Amsterdam, Amsterdam UMC, Amsterdam, The Netherlands; 11 Immunotherapy Laboratory, Department of Medical Oncology, Amsterdam UMC, Cancer Center Amsterdam, Amsterdam, The Netherlands; 12 CimCure BV, Amsterdam, The Netherlands; 13 Department of Pathology, Erasmus MC Cancer Institute, University Medical Center Rotterdam, Rotterdam, The Netherlands

**Keywords:** Angiogenesis, Tumor endothelial cell anergy, ICAM-1, Leukocyte infiltration, Sunitinib

## Abstract

**Purpose:**

Ongoing angiogenesis renders the tumor endothelium unresponsive to inflammatory cytokines and interferes with adhesion of leukocytes, resulting in escape from immunity. This process is referred to as tumor endothelial cell anergy. We aimed to investigate whether anti-angiogenic agents can overcome endothelial cell anergy and provide pro-inflammatory conditions.

**Experimental design:**

Tissues of renal cell carcinoma (RCC) patients treated with VEGF pathway-targeted drugs and control tissues were subject to RNAseq and immunohistochemical profiling of the leukocyte infiltrate. Analysis of adhesion molecule regulation in cultured endothelial cells, in a preclinical model and in human tissues was performed and correlated to leukocyte infiltration.

**Results:**

It is shown that treatment of RCC patients with the drugs sunitinib or bevacizumab overcomes tumor endothelial cell anergy. This treatment resulted in an augmented inflammatory state of the tumor, characterized by enhanced infiltration of all major leukocyte subsets, including T cells, regulatory T cells, macrophages of both M1- and M2-like phenotypes and activated dendritic cells. In vitro, exposure of angiogenic endothelial cells to anti-angiogenic drugs normalized ICAM-1 expression. In addition, a panel of tyrosine kinase inhibitors was shown to increase transendothelial migration of both non-adherent and monocytic leukocytes. In primary tumors of RCC patients, ICAM-1 expression was found to be significantly increased in both the sunitinib and bevacizumab-treated groups. Genomic analysis confirmed the correlation between increased immune cell infiltration and ICAM-1 expression upon VEGF-targeted treatment.

**Conclusion:**

The results support the emerging concept that anti-angiogenic therapy can boost immunity and show how immunotherapy approaches can benefit from combination with anti-angiogenic compounds.

**Supplementary Information:**

The online version of this article contains supplementary available 10.1007/s10456-022-09863-4.

## Introduction

Tumor angiogenesis is a hallmark of cancer progression [1, 2] and mounting evidence demonstrates that it also induces immune suppression and evasion of anti-tumor immunity [3]. Angiogenic factors, such as vascular endothelial growth factor (VEGF) and fibroblast growth factor (FGF), are known to inhibit, directly or indirectly, T cell development and function [4, 5], promote T cell exhaustion through upregulation of immune checkpoints [6, 7], inhibit dendritic cell maturation [8], modulate macrophage polarization [9] and increase the numbers of intratumoral regulatory T cells and myeloid derived suppressor cells [10–12]. Moreover, the tumor vasculature is dysfunctional resulting in insufficient blood perfusion and oxygenation, which leads to tumor hypoxia that has various immunosuppressive effects [13, 14]. Apart from these direct immunosuppressive effects, proangiogenic factors have also been found to hamper leukocyte infiltration by affecting the expression of tumor endothelial adhesion molecules involved in leukocyte extravasation [15–20]. The latter phenomenon was termed tumor endothelial cell anergy, referring to the angiogenesis-induced inability of tumor endothelial cells to respond to inflammatory cytokines, resulting in non-adhesive endothelial surface [15, 21]. Indeed, preclinical studies showed that inhibition of angiogenesis can enhance immune responses through suppression of regulatory T cells [22], reduction of number and function of myeloid derived suppressor cells [23], increase of dendritic cell numbers and activation [24], improvement of T cell responses [25]. Moreover, judicious dosing of anti-angiogenic agents can result in transient normalization of the tumor vasculature physiology [26], which improves tissue oxygenation [27, 28] that might reduce hypoxia-induced immunosuppression. In addition, several preclinical studies demonstrated that tumor endothelial cell anergy could be overcome by anti-angiogenic treatment, resulting in normalized endothelial adhesion molecule expression [29]. The latter is of importance since not only the type of immune cells is relevant for effective anti-tumor immune responses, but also their ability to infiltrate the tumor parenchyma [30]. In addition, adequate tumor immune infiltration improves the response to immune checkpoint inhibition in a variety of cancers [31, 32]. This supports the applicability of anti-angiogenic drugs to improve immunotherapy as these drugs lead to increased expression of endothelial adhesion molecules that are required for trans-endothelial migration of leukocytes into the underlying tumor parenchyma.

Currently, over 90 clinical trials are evaluating the clinical benefit of combining immunotherapy, in particular immune checkpoint inhibition, with anti-angiogenic agents. To date, this led to five FDA approvals for combination therapy in different malignancies [33, 34]. Nevertheless, details on how anti-angiogenic treatment affects the immune infiltrate in human tumors are lacking since treatment with anti-angiogenic drugs is usually in an adjuvant setting. An exception to the latter is renal cell carcinoma (RCC) patients who have received neoadjuvant anti-angiogenic therapy. Here, we performed extensive profiling of the immune infiltrate in primary tumors of RCC patients that received two cycles of VEGF pathway-targeted therapy prior to cytoreductive surgery. We demonstrate that treatment with either sunitinib or bevacizumab stimulates leukocyte infiltration and that overcoming tumor endothelial cell anergy is the mechanism behind these observations. These findings provide important insights into the immunomodulatory effect of anti-angiogenic therapy and contribute to the ongoing debate on the role of angiogenesis inhibitors in immunotherapy.

## Material and methods

### Clinical samples

The clinical samples used for evaluation were primary tumors from patients with metastatic clear cell renal cell carcinoma (RCC), who were treated with either presurgical sunitinib (*n* = 35) or presurgical bevacizumab (*n* = 33). RCC tissues from previously untreated patients were used as controls (*n* = 53). All samples were obtained from retrospective studies or phase II clinical trials, as we previously reported on in earlier papers [35, 36]. The clinical study with preoperative sunitinib treatment was carried out at the Netherlands Cancer Institute, Amsterdam, The Netherlands and is registered under EudraCT 2006–006,491-38 (https://eudract.ema.europa.eu) and at the MD Anderson Cancer Center (MDACC) in Houston, Texas (clinicaltrials.gov identifier: NCT00715442). Patients received a standard therapy of 50 mg sunitinib (2 cycles, 4 weeks on, 2 weeks off therapy) and underwent cytoreductive nephrectomy. Tumor samples obtained from a phase II trial of presurgical bevacizumab completed at the MD Anderson Cancer Center in Houston, Texas (clinicaltrials.gov identifier: NCT00113217) were used. In this single-arm phase II trial, enrolled patients with primary metastatic clear-cell RCC received four doses (10 mg/kg of body weight) of bevacizumab administrated intravenously every 14 days [37].

### Cells and cultures

Human umbilical vein endothelial cells (HUVEC) were isolated from umbilical cords according to standard methods [38]. HUVEC were maintained in RPMI-1640 (Lonza) medium containing 10% fetal bovine serum (Biowest, Nuaillé, France), 10% human serum, 2 mM L-glutamine (Lonza) and 100 μg/mL Pen/Strep (Lonza). For culturing, 0.2% gelatin (Sigma/Aldrich) coated culture flasks were used. For drug treatment of HUVEC as indicated, the cells were harvested with trypsin/ EDTA (Lonza), washed with PBS and seeded at 30.000 cells/well in a 24-well culture plate. Cells were incubated with compounds (see supplementary information) for 48 h.

### Immunohistochemistry

Vascular markers CD31 (DAKO, Glostrup, Denmark) and CD34 (Monosan, Uden, The Netherlands) were used simultaneously for detection of blood vessels, in order to identify nearly all blood vessels [39]. Antibodies to leukocyte markers CD45 (1:100, 2B11, DAKO), CD3 (1:50, F7.2.38, DAKO), CD8 (1A5, Monosan), granzyme B (1:150, GrB-7, Monosan), FoxP3 (1:100, 236A/E7, Abcam, Cambridge, UK), CD83 (1:50, 1H4b, Monosan), CD68 (1:200, 514H12, AbD Serotec, Oxford, UK) and CD163 (1:1000, 10D6, Novacastra Labolatories Ltd, Newcastle, UK) and adhesion molecule ICAM-1 (#4915, Cell Signaling Technology, Danvers, MA, USA) were used. For procedures of standard and fluorescence-based immunohistochemistry see Supplemental Information.

### Immunohistochemical analysis

Microvessel density was determined by 2–4 independent observers in 10 randomly selected fields (200x) [38], and presented as the amount of blood vessels/high power field (HPF, 0.25 mm^2^). The number of ICAM-1^+^ blood vessels was determined by 2 independent observers in 3 randomly selected fields (200x). The amount of tumor-infiltrating leukocytes of each subset was quantified by visual enumeration using bright field light microscopy (Olympus BX50 microscope). Intratumoral leukocytes were counted in 10 randomly chosen high power fields (HPF, 0.25 µm^2^). Due to the low amount of CD83 + positive cells, 15 randomly chosen representative HPFs were counted to increase the reliability. CD68^+^ and CD163^+^ macrophages were scored as 0 (none), 1 (≤ 5%), 2 (5–20%), 3 (20–40%), 4 (40–60%), or 5 (≥ 60%). The ICAM-1 + intensity quantification in the vasculature was performed by analyzing the intensity of 8 symmetrically selected points/vessel using ImageJ software.

### Flow cytometry

Treated HUVEC were harvested and fixed with 1% paraformaldehyde fi 30’. After washing in PBS, unspecific binding sites were blocked with 5% (w/v) BSA in PBS for 30 min at RT. Cells were washed and split in FACS tubes for the staining with monoclonal mouse anti-human ICAM-1 antibody (1:100; clone MEM-111; Monosan). Incubation with antibody was carried out in 50 μl 0.1% (w/v) BSA in PBS at 4 °C for 1 h. Control stainings without primary antibody were carried along for each condition. Detection of bound antibody was performed with allophycocyanin (APC)-conjugated goat anti-mouse Ig (1:100; BD Biosciences) for 30 min at 4 °C for 1 h. Then, samples were washed and transferred and analysed on a FACS-Calibur (Becton & Dickinson). Data analysis was done using FloJo software (Becton & Dickinson).

### RNA isolation and cDNA synthesis

Frozen tissue from a selection of patients was available for RNA isolation, including clear-cell RCC patients pretreated with sunitinib (*n* = 12) and untreated patients as control (*n* = 5). Briefly, frozen tissue was lysed with TRIzol (Invitrogen, Lucern, Switzerland) and RNA was extracted with NaAC 4.5 pH (3 M), acidic phenol 4.5 pH (AM9720, Ambion Inc, Austin, TX, USA) and chloroform (0.1:1:0.2). RNA concentrations were measured using the NanoDrop-2000 Spectrophotometer (Thermo Scientific, Wilmington, DE, USA). One μg of total RNA was incubated for 5 min at 70 °C, and cDNA synthesis was performed for 1.5 h at 42 °C with 400 U of M-MLV reverse transcriptase RNase H (Promega, Leiden, the Netherlands) in 20 µl of 1 × first strand buffer (Promega), and 1 mM dNTPs in the presence of 10 U RNase inhibitor rRNasin (Promega) and 0.5 µg random primers (Promega). The reverse transcriptase activity was inactivated by incubation at 95 °C for 5 min and following addition of 1xTE up to a final volume of 50 µL the cDNAs were stored at -20 °C.

### Real-time quantitative PCR

For real-time quantitative RT-PCR (qPCR) primers targeted against the reference genes β-actin, cyclophilin A, and β-2 microglobulin were used, in addition to primers targeted against the mouse and human intercellular adhesion molecule 1 (ICAM-1), vascular cell adhesion molecule 1 (VCAM-1) [38], and mouse E-selectin (See Suppl. Table S1). Primer design and qPCR were performed as described previously [40].

### Trans-endothelial migration assay

Transwell inserts (3 µm pore diameter, Costar) were placed in 24-well plates, seeded with 15.000 HUVEC and cultured overnight in 200 µl of culture medium (and 600 µl of culture medium in the lower compartment). On day 2, angiogenesis inhibitors were added to both upper and lower compartments. After 72 h cells were washed and new medium without inhibitors was added. Subsequently, peripheral blood mononuclear cells (PBMCs), isolated through Ficoll-Hypaque density gradient centrifugation of freshly drawn blood, were added to the upper compartment of the transwell system and incubated for 16 h. Spontaneous and SDF1α-stimulated transmigrated leukocytes were harvested from the lower compartment and counted. Adherent cells were quantified by microscopy of the outer bottom of the inserts. To stain the adherent cells, the inserts’ inside were washed and cleaned with cotton wool sticks, to remove HUVECs and non-migrated leukocytes. Inserts were then placed in 24-well plate wells with 600 µl of crystal violet for 3 min. Subsequently, inserts were washed twice with PBS and then put upside-down under a regular microscope for cell counting. Ten representative images of 1 mm^2^ areas of the insert were taken and counting of the number of adherent cells in each picture was done manually with Adobe Photoshop.

### RNA sequencing

RNAseq count data were processed in RStudio 1.1.456 (R 3.5.1) using DESeq2 according to standard pipelines^6^. Based on unsupervised clustering of the samples, 1 control sample and 2 sunitinib-treated samples were left out of the analysis as they deviated considerably. Differentially expressed genes (fold change > 2) between untreated (*n* = 4) and sunitinib-treated (*n* = 9) patient samples were obtained at an adjusted *p* value (padj) < 0.01 (*n* = 1119; 653 upregulated and 466 downregulated in sunitinib-treated) and padj < 0.05 (*n* = 2236; 1215 upregulated and 1021 downregulated in sunitinib-treated). Differentially expressed genes (at padj < 0.05) were analyzed using DAVID (https://david.ncifcrf.gov/) to identify enrichment in gene ontologies (biological process and KEGG pathways). A selection of enriched ontologies was made based on the number of genes per group (> = 10) and (padj < 0.01 for sunitinib-induced and padj < 0.05 for untreated induced) which were subsequently manually clustered into biologically relevant groups. Individual genes represented in these enriched ontology clusters were subject to protein interaction analysis using STRING (https://string-db.org). To keep data presentation manageable, for the immune regulation genes cluster only those that displayed a fold change of > 4 in sunitinib treated RCC vs untreated were taken along (*n* = 148). Confidence levels were set to 0.9 (highest confidence) and for presentation, small clusters and unconnected nodes were removed.

### Cibersort analysis

Cibersort [41] (https://cibersort.stanford.edu/) was run online using default recommend settings. Since a number of the 22 defined immune subsets returned no or very little presence, the categories were simplified by merging a number of functionally related immune subsets.

### Statistical analysis

All obtained data were verified for normal distribution by D ´Agostino Test. Non-parametric Kruskal–Wallis and MannWhitney U as post hoc test were used to determine statistical significance for data obtained in the infiltration study. Corresponding correlations were analyzed using non-parametric Spearman correlation test. For statistical analysis of mRNA and protein expression data an unpaired Student's t test was applied. All statistical analyses were done with GraphPad Prism version 5.01 for Windows (GraphPad Software Inc., La Jolla, CA, USA). Values of *p* < 0.05 were considered statistically significant (*p < 0.05, **p < 0.01, ***p < 0.001).

## Results

### Targeting the VEGF signaling axis inhibits vessel density, induces leukocyte infiltration and generates an inflammatory profile in renal cell carcinoma tissues

**Fig. 1:**
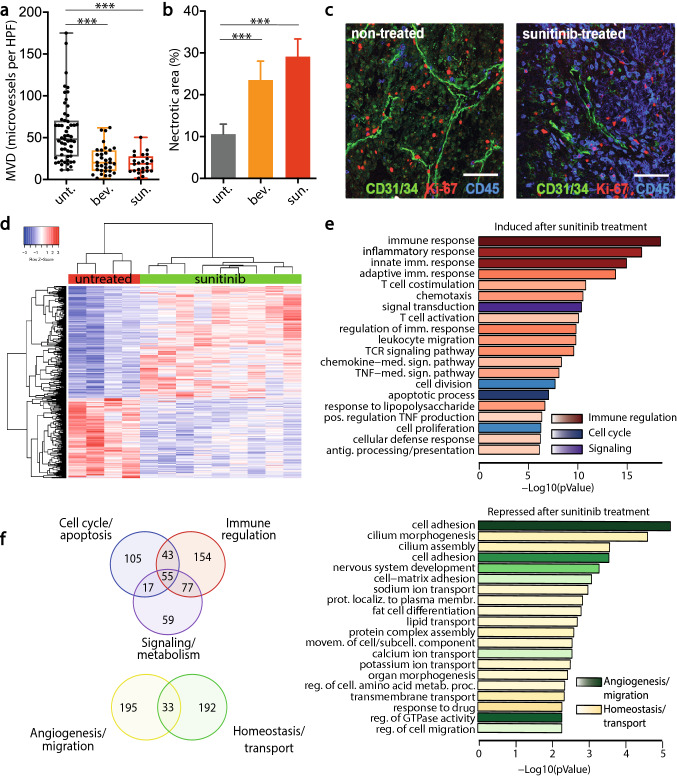
VEGF axis targeting inhibits vessel density, induces leukocyte infiltration, and generates an inflammatory profile in renal cell carcinoma tissues. **a.** Microvessel density counts in CD31/34-stained sections of RCC tumors from untreated patients (*n* = 53) and patients treated with 2 cycles of bevacizumab- (*n* = 33) or sunitinib (*n* = 24) monotherapy prior to cytoreductive surgery. Scatter plots show numbers of microvessels per high-power field (HPF, 0.25 mm^2^) for each patient. Median values are indicated, boxes extend from the 25th to 75th percentiles and the whiskers extend from the minimum to the maximum values. Statistically significant differences are indicated by asterisks (***p < 0.001), as compared to the untreated control group. **b.** Mean areas of necrosis, indicated by percentage of the complete tumor section, ± SEM, ***p < 0.001) as compared to the untreated control tissues. **c.** Representative confocal immunofluorescence microcopy images with CD31/34 (blood vessels, green), Ki-67 (proliferation marker, red) and CD45 (leukocytes, blue) staining of tissues from non-treated and sunitinib-treated RCC patients. Scalebar represents 50 µm. **d.** Heatmap of significantly differentially expressed genes (fold-change > 2 and adj. p-value < 0.01) between untreated and sunitinib-treated RCC. **e.** Top: Significantly enriched gene ontologies (GO, Biological Process) for genes upregulated in sunitinib-treated RCC. Bars are color-coded according to three prevalent functional clusters. Lengths of the bars are indicative of p-values for enrichment whereas color intensity is related to the maximum number of genes present in the denoted GO term. Bottom: Enriched gene ontologies (GO, Biological Process) for genes downregulated in sunitinib-treated RCC. **f.** Top: Venn diagram of overlap of sunitinib induced genes in different clusters of enriched gene ontologies. Bottom: Venn diagram of overlap of sunitinib repressed genes in different clusters of enriched gene ontologies

To investigate the effect of anti-angiogenic therapy on leukocyte infiltration into tumors, primary renal cell carcinoma (RCC) tissues were obtained from patients treated with neoadjuvant VEGF-targeted therapy [35, 37, 42]. Treatment with two cycles of either sunitinib or bevacizumab resulted in a statistically significant decrease in microvessel density (*p* < 0.001, Fig. 1a). This was accompanied by increased areas of necrosis in the tumor (*p* < 0.001, Fig. 1b). In the viable areas triple fluorescence staining showed, next to inhibition of angiogenesis (CD31/CD34) and proliferation of both tumor- and endothelial cells (Ki-67), a marked increase of the number of infiltrating leukocytes (CD45, Fig. 1c), which suggests a correlation with induction of necrosis.

Further analysis of the tumors in these patient groups through RNA sequencing of untreated and sunitinib-treated RCC tissues was performed. Over 1000 genes were differentially expressed by more than twofold (Fig. 1d). It was noted that within the overexpressed genes in the sunitinib-treated group biological processes associated with immune function, signaling/metabolism and cell cycle/apoptosis regulation were highly enriched (Fig. 1e, top panel), whereas overexpressed genes in the control (untreated group) relative to the sunitinib-group were mainly associated with tissue homeostasis, such as transport and protein metabolism, as well as cell migration and matrix adhesion that are essential for angiogenesis (Fig. 1e, bottom panel). Overlapping enrichment of individual biological process ontologies for genes overexpressed or downregulated in sunitinib-treated samples are shown in Fig. 1f. Protein–protein interaction analysis was performed to investigate functional connections between differentially expressed genes. Different protein clusters were observed, which showed that sunitinib treatment induced a major subset of chemokines and their receptors, as well as CD8 and interleukins (Suppl. Figure S1a-b). Furthermore, sunitinib treatment repressed the expression of interacting proteins responsible for extracellular matrix (ECM) binding through collagens and integrins, angiogenic signaling through angiopoietins and angiogenic growth factors, and signaling through Rho GTPases and DLL/Notch. Taken together, these data support the view that sunitinib treatment simultaneously represses angiogenesis and stimulates immune function.

### Immune infiltration is increased by VEGF pathway-targeted treatment

**Fig. 2:**
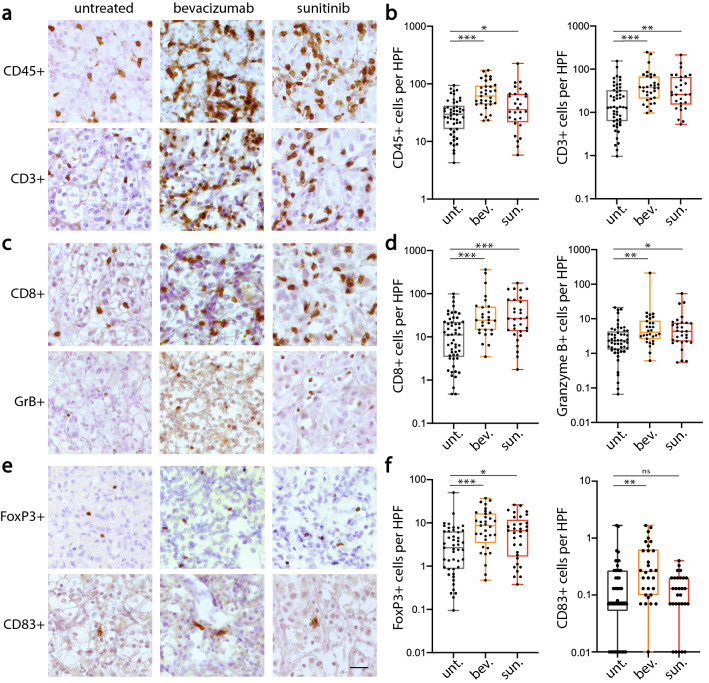
Enhanced infiltration of leukocyte subsets in RCC tumor tissues after pre-surgical treatment with VEGF-targeted therapy. Immunohistochemical detection of leukocyte subsets in RCC tissues of bevacizumab- (*n* = 27–33) and sunitinib (*n* = 27–33) treatment groups and untreated controls (*n* = 49–53). Stainings (brown) for total leukocytes and T lymphocytes (CD45^+^, CD3^+^, panel **a**), total cytotoxic T cells (CD8^+^) and activated granzyme B^+^ CD8^+^ T cells (panel **c**), regulatory T cells (FoxP3^+^) and mature CD83^+^ dendritic cells (panel **e**). Quantification of the intratumoral leukocyte subsets (panels **b**, **d** and **f**) is shown as number of cells per high-power field (HPF, 0.25 mm^2^). Median values are indicated, boxes extend from the 25^th^ to 75th percentiles and the whiskers extend from the minimum to the maximum values. Statistically significant differences are indicated by asterisks (*p < 0.05, **p < 0.01, ***p < 0.001), as compared to the untreated control group. ns = not significant. The scale bar in E represents 10 µm and is valid for all photomicrographs

To investigate the effect of blocking VEGF signaling on the composition of the immune infiltrate, a quantitative assessment of the different leukocyte subtypes was performed by immunohistochemical staining. While we observed a large variation in the total amount of infiltrated leukocytes in the untreated RCC tissues, a statistically significant increase of total leukocyte infiltration (CD45^+^), as well as of T lymphocytes (CD3^+^), was measured in RCC tissues after treatment with both types of VEGF-targeted therapy (Fig. 2a, b). The same was observed for CD8^+^ cytotoxic T lymphocytes, which increased by 2–threefold in both the bevacizumab (*p* < 0.001) and sunitinib (*p* < 0.001) treatment groups (Fig. 2c-d). The increased infiltration was also observed for the fraction of activated cytotoxic cells, as assessed by detection of granzyme B (Fig. 2c-d). Interestingly, similar significant responses were observed in the numbers of FoxP3^+^ regulatory T lymphocytes (Fig. 2e, f). Regarding CD83^+^ mature dendritic cells, only low numbers were detected that did significantly increase (*p* < 0.01) in the bevacizumab treatment group, while in the sunitinib-treated patients the numbers were not altered (Fig. 2f).

### VEGF-targeted therapy increases macrophage infiltration in primary RCC tissue.

**Fig. 3:**
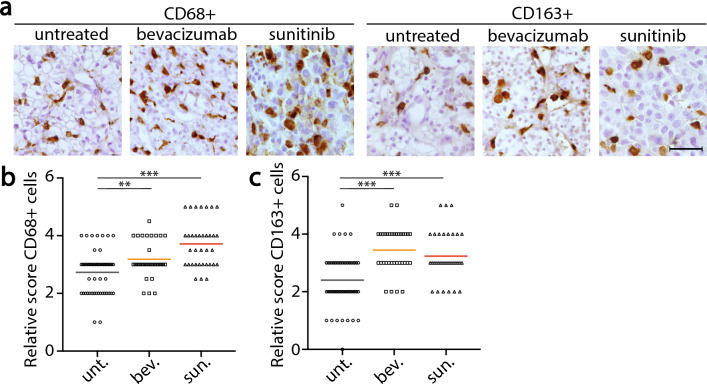
Enhanced infiltration of macrophages after VEGF-targeted therapy. **a.** Immunohistochemical detection of the total number of macrophages (CD68^+^) and the number of M2-like CD163^+^ macrophages in primary RCC tumor sections from patients treated with bevacizumab (*n* = 33) or sunitinib (*n* = 35) prior to cytoreductive surgery, as well as from untreated control tissues (*n* = 53). Scale bar represents 50 µm. Scatter plot quantifications of relative numbers of infiltrated CD68^+^ macrophages (**b**) and the subpopulation of M2-like CD163^+^ macrophages (**c**). Horizontal bars indicate the mean values for each patient group. Significance is indicated by asterisks (**p < 0.01 and ***p < 0.001)

Finally, we evaluated the macrophage infiltration and observed that these cells represent a large fraction of tumor infiltrating leukocytes in RCC (Fig. 3a). Bevacizumab and sunitinib treatment of patients with RCC induced an increase in the total numbers of CD68^+^ macrophages in the primary tumors (*p* < 0.01 and *p* < 0.001 respectively, Fig. 3a-b), associated with the induction of necrosis, shown in Fig. 1b. Furthermore, tumors in both treatment groups contained significantly enhanced numbers of CD163^+^ M2 macrophages (*p* < 0.001, Fig. 3a-c).

To substantiate these observations, Cibersort analysis was performed [41]. This tool, referred to as digital flow cytometry, allows estimation of the relative abundance of immune cell subsets in complex samples based on bulk gene expression data of multiple lineage specific genes [43]. RNAseq data of the RCC tissues were used to estimate the presence of different immune subsets (Suppl. Figure S1c). Although Cibersort provides only relative data, a significant increase in the fraction of infiltrating CD8^+^ cytotoxic T cells after sunitinib-treatment was confirmed (Suppl. Figure S1d). This analysis also confirmed that macrophages make up a large fraction of both treated and untreated RCC and that the predominant subtype is the M2-like phenotype. This digital flow cytometry analysis further corroborated our observations that VEGF targeting treatment promotes immune infiltration.

### Anti-angiogenic compounds induce endothelial ICAM-1 expression in vitro

**Fig. 4:**
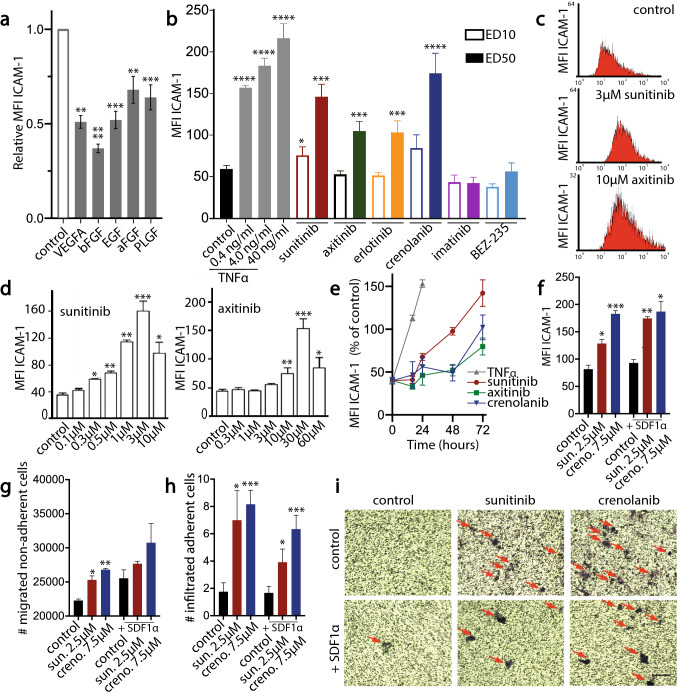
Induction of endothelial ICAM-1 expression by angiogenesis inhibitors in vitro; impact on trans-endothelial migration of leukocytes. **a.** Suppression of endothelial ICAM-1 expression by VEGFA (20 ng/ml) and other angiogenic growth factors (bFGF, 10 ng/ml; EGF, 40 ng/ml; aFGF, 10 ng/ml; PLGF, 50 ng/ml. Results are presented as mean ICAM-1 fluorescence intensity, ± SEM, *n* = 4–6). **b.** Induction of endothelial ICAM-1 expression by ED_10_ and ED_50_ doses of a selection of angiostatic compounds targeting a variety of signaling pathways (sunitinib 0.5 and 2.5 µM; axitinib 1 and 10 µM; erlotinib 2 and 20 µM; crenolanib 2 and 7.5 µM; imatinib 1 and 5 µM; BEZ-235 0.005 and 0.02 µM, respectively, see Suppl. Table S2), relative to a range of concentrations of the positive control TNFα (0.4–40 nM). Results are expressed as the mean fluorescence intensity, ± SEM, *n* = 3. **c.** Flow cytometric analysis of surface ICAM-1 expression on human umbilical vein endothelial cells (HUVEC) exposed for 72 h to 3 µM sunitinib or 10 µM axitinib. **d.** Dose-dependence of endothelial ICAM-1 induction upon treatment with sunitinib and axitinib. **e.** Time-dependence of ICAM-1 induction by ED_50_ doses of drugs. Upregulation of ICAM-1 (**f**) and resulting induction of spontaneous and SDF-1α-induced (200 ng/ml) transendothelial migration of non-adherent (**g**) and adherent (**h**) cells. **i.** Images of transmigrated adherent cells on the trans-well inserts. Significance in all panels is indicated by asterisks, *p < 0.05, **p < 0.01, ***p < 0.001, ****p < 0.0001

To explore the role of endothelial cell anergy in the increased immune infiltration after anti-angiogenic treatment, we focused on ICAM-1, a key molecule in facilitating leukocyte endothelial transmigration. In line with our previous work, we confirmed that VEGFA and other angiogenic growth factors suppress ICAM-1 expression in endothelial cells (Fig. 4a). Importantly, subsequent treatment of HUVEC, in which angiogenic signalling was induced by culture in human serum, with sunitinib and several other anti-angiogenic drugs counteracted tumor endothelial cell anergy, as exemplified by induction of endothelial ICAM-1 expression in vitro, both at the mRNA- and protein levels (Fig. 4b-c). In these studies, bevacizumab was not included, because of its indirect effect on endothelial cells, by exogenous VEGF neutralization. The anti-angiogenic agents were tested at their ED_10_ and ED_50_ doses (effective doses where 10% and 50% of maximal proliferation inhibition is observed, respectively, Fig. 4b). Interestingly, two inhibitors of endothelial cell proliferation, imatinib (targeting PDGF receptors) and BEZ-235 (an mTOR inhibitor), did not affect ICAM-1 expression (Fig. 4b), indicating that the observed effect is not merely an epiphenomenon of endothelial cell growth arrest. Of note, the level of upregulation of ICAM-1 by anti-angiogenic drugs was approximately 60–90% of the response to the control inflammatory cytokine TNFα at 40 ng/ml (Fig. 4b). Similar results were observed in the immortalized endothelial cell line ECRF24 (Suppl. Figure S2). Additional experiments indicated that the ICAM-1 induction was dose- (Fig. 4d) and time dependent (Fig. 4e).

### Anti-angiogenic compounds stimulate transendothelial migration of leukocytes.

To investigate whether the induction of ICAM-1 expression controls leukocyte trafficking, trans-well migration of peripheral blood mononuclear cells over an endothelial cell monolayer was analyzed. Exposure of the endothelial cells for 72 h to either sunitinib or crenolanib prior to migration slightly but significantly increased the numbers of transmigrated non-adherent cells (Fig. 4g). A more profound effect was observed for adherent cells of monocytic origin (Fig. 4h-i). The presence of the chemotactic cytokine SDF1α in the lower compartment increased the transmigration of non-adherent cells but masked the effect of the angiostatic compounds (Fig. 4g). In adherent cells, SDF1α had some reducing effect but angiostatic compounds still significantly stimulated transendothelial migration (Fig. 4h-i). Of note, SDF-1α had no significant effect on the endothelial ICAM-1 expression (Fig. 4f), indicating that the increased transendothelial migration was an effect of increased ICAM-1 expression per se, rather than of chemotaxis.

### Anti-angiogenic therapy induces endothelial adhesion molecules in primary RCC tissue

**Fig. 5:**
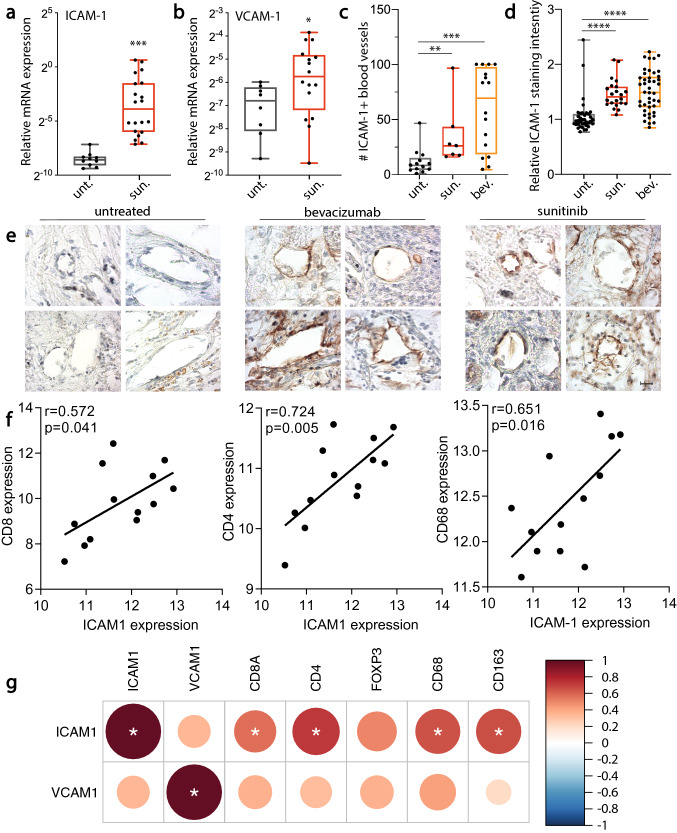
Anti-angiogenic therapy induces endothelial adhesion molecules in primary RCC tissue. **a**, **b** qPCR analysis of ICAM-1 and VCAM-1 expression in sunitinib-treated and untreated primary RCC tissues. **c** Quantification of the number of ICAM-1^+^ vessels in tumor tissues of treated (sunitinib *n* = 7; bevacizumab *n* = 16) and untreated RCC patients (*n* = 12). **d** Analysis of ICAM-1 expression density in a selection of microvessels of treated (sunitinib *n* = 22; bevacizumab *n* = 42) and untreated RCC tissues (*n* = 34), examples of blood vessels in **e** (scale bar in lower right image represents 10 µm). **f**, **g**. Correlation of gene expression based on RNAseq data of all tumors. **f** Correlation plots of individual samples for ICAM-1 versus CD8, CD4 and CD68 expression. **g** Correlation heatmap plot for all samples. Color legend indicates the Pearson correlation coefficient. Significance in all panels, unless specifically indicated, is indicated by asterisks *p < 0.05, **p < 0.01, ***p < 0.001

To further investigate the expression of endothelial adhesion molecules during VEGF-targeted therapy, a preclinical xenograft mouse model of RCC was used. Human Caki-1 RCC cells were injected subcutaneously in the flank of athymic Foxn1^nu^ mice. When palpable tumors were present, mice were randomized in two groups. One group of mice (*n* = 5) was treated with sunitinib, the other (*n* = 5) with vehicle. Tumor growth curves show significant inhibition of tumor growth, with no effect on body weight (Suppl. Figure S3a-b, respectively). Subsequent gene expression analysis of the excised tumor tissues demonstrated significantly increased vascular Icam-1, Vcam-1 and E-selectin gene expression (Suppl. Figure S3c). To assess whether a similar regulation occurs in human tumor tissues after sunitinib-treatment, a set of primary RCC tissues was subjected to ICAM-1 expression analysis by qPCR and immunohistochemistry. This revealed that sunitinib-treatment resulted in a significant induction of ICAM-1- and VCAM-1 mRNA expression (Fig. 5a-b). Analysis of ICAM-1 expression at the protein level by the enumeration of ICAM-1^+^ blood vessels, also showed a significant increase (Fig. 5c). Furthermore, quantification of ICAM-1 expression levels by density analysis showed similar effects (Fig. 5d-e). These results were also observed in bevacizumab-treated patients (Fig. 5c-e). Importantly, the enhanced adhesion molecule expression correlated with increased infiltration of CD8 + , CD4 + , and CD68 + leukocytes based on RNAseq analyses (Fig. 5f-g). As expected, correlations for VCAM-1 showed a similar trend but were not significant (Fig. 5g), given the relatively modest contribution of VCAM-1 in leukocyte transmigration [44]. Altogether, the presented results support that VEGF-targeted treatment can counteract tumor endothelial cell anergy and promote the formation of an inflammatory tumor infiltrate.

## Discussion

In this study we provide evidence that anti-angiogenic therapy by targeting the VEGF signaling axis can counteract tumor endothelial cell anergy in renal cell carcinoma (RCC). Using preclinical models and RCC patient tumor tissues, we showed that anti-angiogenic treatment induced pro-inflammatory activity. This included enhanced leukocyte infiltration in the tumor tissue, confirming the results by Liu et al. [45]. Moreover, angiostatic drugs induce a dose- and time-dependent normalization of endothelial adhesion molecule expression, in particular of ICAM-1 which is necessary as well as sufficient for leukocyte infiltration [44]. Although these observations leave room for the fact that infiltrated leukocytes may contribute to the vascular normalization, this is unlikely since the regulation of adhesion molecules is also observed in cultures of isolated endothelial cells (Fig. 4b and c). Many previous studies have addressed the importance of ICAM-1 in leukocyte extravasation using ICAM-1 blocking antibodies [46]. The evidence for angiostasis-induced leukocyte infiltration provides a rational for the current success of treatment strategies that combine angiogenesis inhibitors and immunotherapy [3, 47]. In this field > 90 clinical trials, mostly involving immune checkpoint inhibitors, are currently ongoing and to date 5 combination therapies have been FDA approved for treatment of RCC [34, 48], non-small cell lung cancer [33] and hepatocellular carcinoma [49].

Angiogenesis inhibition for the treatment of cancer is still an attractive strategy, but current drugs have only limited effects on patient survival, even while mostly in combination with other (non-immune) treatment modalities. This is due to drug resistance, as most angiostatic drugs target tumor produced growth factor signaling axes and tumor cells rapidly evolve mechanisms to switch to other angiostimulatory signaling pathways [50–52]. Patients with RCC have long been preferred for testing angiostatic drugs because of treatment resistance to conventional drugs and high vascular indices. Consequently, RCC is often the selected tumor type for mechanistical studies on the effects of angiogenesis inhibitors. Hence, we analyzed tumor tissues from RCC patients who were treated with sunitinib or bevacizumab prior to cytoreductive surgery. While the main aim of the studies was to investigate whether neoadjuvant anti-angiogenic treatment contributed to patient survival, the study setup provided a unique opportunity to investigate the proinflammatory activity of anti-angiogenic drugs and the role of tumor endothelial cell anergy. We propose the latter to be a vascular immune checkpoint, as the main traits of immune checkpoints are paralleled. Both immune checkpoints and the vascular counterpart are (i) immune modulatory pathways maintain immune homeostasis, (ii) providing immune escape when hijacked by tumors, and (iii) therapeutically actionable targets to improve anti-tumor immunity.

Endothelial cell anergy is a rather robust mechanism dictated by tumor cells to compromise immunity by prevention of leukocyte adhesion [18]. Indeed, normalization of endothelial cell adhesiveness by angiogenesis inhibitors, does not only stimulate entry into the tumor of anti-tumor immune cells such as cytotoxic T lymphocytes [29, 53], but also immune cells with pro-tumor activity, such as regulatory T cells and macrophages with M2-like phenotype. However, it is widely known that enhanced infiltration is associated with longer survival so it is assumed that the infiltrated anti-tumor leukocytes outperform the pro-tumor infiltrate. Enhanced infiltration of cytotoxic T cells and macrophage subsets was demonstrated by immunohistochemistry and Cibersort. It is important to note that the technique used here, i.e. single color immunohistochemistry, may potentially lead to incorrect- or overinterpretation of the data. Indeed, for Foxp3 it is known that expression is not limited to CD4^+^ regulatory T cells (Tregs). However, the detection threshold by IHC is such that the majority of stained FoxP3^+^ cells will in fact be activated Tregs, that express FoxP3 at higher levels than other cell types. FoxP3 single stains have therefore been regularly used to quantify Tregs by immunohistochemistry in tumors [54, 55]. The predominant nuclear staining of FoxP3 further confirms the detected FoxP3^+^ cells to be activated Tregs. CD163 can indeed also be expressed by dendritic cells (DC), but in tumors is then also often induced by IL-10 and may signal a suppressed/immune suppressive M2-like functional state in these DCs that like macrophages display a high phenotypic plasticity. Moreover, DCs (including the recently described CD163^+^ cDC3) are far less numerous than CD163^+^ macrophages in the tumor microenvironment. The high density of CD163^+^ cells with a macrophage morphology makes it most likely that these represent M2-like TAMs. CD83 may be expressed by B cells (at low frequencies) but is more generally recognized as a DC maturation marker. Indeed, the morphology of the CD83^+^ cells in our study is consistent with that of DCs. Finally, Granzyme-B may also be expressed by NK cells, but these are hardly found infiltrating human solid tumors. The observed Granzyme B^+^ cells are therefore most likely T cells with cytotoxic potential. Cibersort is designed to deconvolute bulk expression data into prediction of numbers of different infiltrating cells in tissues. Cibersort is therefore a prediction tool, based on previous definitions of immune cells and their markers and in principle, does not solve the problem associated with single color IHC. Nevertheless, Cibersort analysis reveals an increase of Tregs after sunitinib treatment, which is paralleled by an increase in FoxP3 expression at the RNA level, which is in concordance with the IHC data, therefore strengthening the IHC data. Likewise, sunitinib treated tissues showed moderately increased RNA levels of CD83 and CD163.

The results described in this study are the first to demonstrate in patients that the proinflammatory activity of anti-angiogenic drugs is based on overcoming tumor endothelial cell anergy. The clinical results are observed for intervention in the VEGF signaling axis, but in vitro data show similar results for targeting other growth factor receptor pathways, suggesting that treatment with other anti-angiogenic drugs will similarly overcome endothelial anergy. Mechanistically, we demonstrated that the anergic state of tumor endothelial cells is not an epiphenomenon of the proliferative state of the cells, as two anti-proliferative drugs, i.e. imatinib and BEZ-235, were found not to normalize endothelial ICAM-1 expression. Therefore, it is assumed that overcoming endothelial cell anergy is mediated by direct metabolic [56] and epigenetic regulatory events [57] in the targeted endothelial cells. There are multiple other possible scenarios behind the increased inflammatory microenvironment boost post antiangiogenic therapy. A recently published study by Ferician et al. suggests the role of JAK/STAT pathway in defining a special subgroup of ccRCC with moderate and high VEGF expression [58]. Chang et al. described the role of JAK/STAT pathway in cancer associated inflammatory microenvironment [59]. Additionally, one could question why angiogenic growth factors such as VEGF force the anergic state in tumor endothelial cells. We hypothesize that endothelial cell anergy is copied from a developmental gene expression profile in embryonic and fetal stages, where leukocyte infiltration is prevented to ensure proper development of organs. We think it is a plausible explanation as it is shown here that the effect is shared with other angiogenic growth factors. Likewise, as demonstrated here, not only drugs targeting the VEGF axis can overcome tumor endothelial cell anergy, also drugs targeting epithelial growth factor receptor (EGFR, erlotinib) and platelet derived growth factor receptor (PDGFR, crenolanib) can normalize ICAM-1 expression.

While it seems beneficial to increase leukocyte infiltration, the immunosuppressive tumor microenvironment may still dictate conditions of suppressed immunity. Therefore, a clear application of the current study is the combination of anti-angiogenic drugs with immunotherapy strategies that overcome immune checkpoint expression. It is known that tumors infiltrated by leukocytes respond better to immune checkpoint inhibitors. Tumors with an immune excluded phenotype, i.e. leukocyte infiltration only present in the tumor stromal areas, respond less well, whereas tumors which are immunological deserts, i.e. low or no infiltration throughout the tumor, are the least likely to respond [60]. It is expected that these low infiltrated tumors can be enhanced to the responsive level by treatment with anti-angiogenic compounds. Figure 2 demonstrates that tumors of a number of patients contain very low numbers of CD3 (< 2 cells/HPF) and CD8 (< 2 cells/HPF) cells. Interestingly, none of the tumors showed such low infiltration in either of the treated groups. The current results may therefore also be used for treatment decisions on scheduling of the combination therapy. It would seem most effective when angiostatic treatment precedes the immunotherapy arm of the treatment. This would also preclude any negative effects that the anti-angiogenic drugs may have on the effector immune cells. Although it may be considered counterintuitive to inhibit vascularization to increase the number of immune cells in the tumor, it should be realized that the regulation of endothelial adhesion molecules is a fast process and can be accomplished over a period of hours, while measurable reductions in blood vessels takes weeks. In this context it would be interesting to investigate whether endothelial anergy can be overcome with low-dose treatment with anti-angiogenic drugs that normalizes vessel adhesiveness but does not reduce the number of blood vessels.

In conclusion, we demonstrated that one of the underlying mechanisms explaining the current success of the combination of anti-angiogenic- and immunotherapy is overcoming the phenomenon of tumor endothelial cell anergy by the angiostatic arm of the therapy. The current results will have major impact on the future success of cancer treatment.

## Supplementary Information

Below is the link to the electronic supplementary material.

Electronic supplementary material 1 (DOCX 724 kb)
