# Supplementary material for: Proinflammatory activity of VEGF-targeted treatment through reversal of tumor endothelial cell anergy
Source: Angiogenesis. 2022 Dec 2;26(2):279–93. doi: 10.1007/s10456-022-09863-4 (PMC10119234; doi:10.1007/s10456-022-09863-4)
Supplement: Supplementary file 1 — Electronic supplementary material 1 (DOCX 724 kb) [file 10456_2022_9863_MOESM1_ESM.docx]

**Supplemental information for:**

**Proinflammatory activity of VEGF-targeted treatment through**

**reversal of endothelial cell anergy**

Patrycja Nowak-Sliwinska^1,2,3 #^ *, Judy R. van Beijnum^1 #^, Christian J. Griffioen^1^,

Zowi R. Huinen^1^, Nadine Grima Sopesens^1^, Ralph Schulz^1^, Samir V. Jenkins^4^,

Ruud P.M. Dings^4^, Floris H. Groenendijk^5^, Elisabeth J.M. Huijbers^1^, Victor L.J.L. Thijssen^1^,

Eric Jonasch^6^, Florry A. Vyth-Dreese^7^, Ekaterina S. Jordanova^9^, Axel Bex^8^,

René Bernards^5^, Tanja D. de Gruijl^10^, Arjan W. Griffioen^1^*

^1^Angiogenesis Laboratory, Department of Medical Oncology, Amsterdam University Medical Centers, Vrije Universiteit Amsterdam, Cancer Center Amsterdam, Amsterdam, The Netherlands; ^2^School of Pharmaceutical Sciences, Faculty of Sciences, University of Geneva, Switzerland; ^3^Institute of Pharmaceutical Sciences of Western Switzerland, University of Geneva, Geneva, Switzerland; ^4^Department of Radiation Oncology, University of Arkansas for Medical Sciences, Little Rock, AR, USA; ^5^Division of Molecular Carcinogenesis, Oncode Institute, The Netherlands Cancer Institute, Plesmanlaan 121, Amsterdam, 1066, CX, The Netherlands; ^6^Department of Genitourinary Oncology, MD Anderson Cancer Center, Houston, TX, USA; ^7^Division of Immunology and ^8^Department of Urology, The Netherlands Cancer Institute, Amsterdam, The Netherlands; ^9^Center for Gynaecologic Oncology Amsterdam, Amsterdam University Medical Centers, Vrije Universiteit Amsterdam, The Netherlands; ^10^Immunotherapy Laboratory, Department of Medical Oncology, Amsterdam University Medical Centers, Vrije Universiteit Amsterdam, Cancer Center Amsterdam, Amsterdam, The Netherlands.

*** Correspondence to:**

Prof. dr. Arjan W. Griffioen, Angiogenesis Laboratory, Department of Medical Oncology, Amsterdam University Medical Center, Vrije Universiteit Amsterdam, Cancer Center Amsterdam, Amsterdam, The Netherlands, Phone: +31-20-4443374, ORCID: [0000-0002-8508-4754](https://orcid.org/0000-0002-8508-4754),

**E-mail:** [a.griffioen@amsterdamumc.nl](mailto:a.griffioen@amsterdamumc.nl)

Prof. dr. Patrycja Nowak-Sliwinska, Rue Michel-Servet 1, CMU, 1211 Geneva 4, Switzerland, Phone: +41 22 3793352, ORCID : [0000-0002-8299-0444](https://orcid.org/0000-0002-8299-0444),

**E-mail:** [Patrycja.Nowak-Sliwinska@unige.ch](mailto:Patrycja.Nowak-Sliwinska@unige.ch)

**This PDF file includes:**

Supplementary methods

Figures S1 - S3

Tables S1 and S2

**Supplementary Methods**

*Cells and cultures*

Caki-1 cells were cultured in RPMI-1640 (Lonza) medium containing 10% fetal bovine serum (Biowest), 2mM L-glutamine (Lonza) and 100 μg/mL Pen/Strep (Lonza). Anti-angiogenic agents used included sunitinib (Pfizer Inc, New York, NY, USA), axitinib (LC laboratories, Woburn, MA, USA ), erlotinib (LC laboratories), crenolanib (Selleck Chemicals, Munich, Germany) and imatinib (LC laboratories) and BEZ-235 (Chemdea LCC, Ridgewood, USA). The concentrations used for each drug were selected to be the effective dose inhibiting the cell viability for 10% (ED_10_) or 50% (ED_50_).

*Immunohistochemistry*

Paraffin sections (5 μm thickness) were deparaffinized with xylene and rehydrated in ethanol. Endogenous peroxidase activity was blocked using 3% H_2_O_2_ in methanol for 15 minutes at room temperature. Antigen retrieval was achieved with Tris-EDTA buffer (10 mM Tris- 1mM EDTA, pH 8) incubation and microwave heating for 15 minutes. Subsequently, the slides were blocked for 30 minutes with 0.5% BSA in PBS. Primary antibodies against the above mentioned vascular and leukocyte markers were applied for one hour, and after 3 washings incubated with horseradish peroxidase (HRP)-conjugated secondary antibodies for 30 minutes. HRP-conjugated rabbit anti-mouse IgG (1:100; Dako) was applied for the stainings of CD3, CD8, CD83 as well as granzyme B and the BrightVision HRP-conjugated goat anti-mouse IgG (ready-to-use; ImmunoLogic) for CD68, CD163 and FOXP3 stainings. Staining was developed with diaminobenzidine (DAB, Sigma-Aldrich, Steinheim, Germany). For detection of proliferative cells, a rabbit-polyclonal anti-Ki-67 (Neomarker) antibody was used, followed by a peroxidase labelled polyclonal swine anti-rabbit IgG (DAKO). The slides were counterstained using hematoxylin (Sigma-Aldrich). Mounting was performed with Quick-D mounting medium (Klinipath). Each staining included a negative control for each sample applying secondary without primary antibody. Between all incubation steps, slides were extensively rinsed with PBS with or without 0.05% Tween.

The triple immunofluorescence staining was performed as described previously {Griffioen, 2012 #813}. Cryosections fixed in acetone were pre-incubated in 5% (v/v) normal goat serum (Sanquin, Amsterdam, The Netherlands) for 30 minutes. Next, the samples were incubated in a mixture of mouse anti-CD31 (1:100, DAKO), mouse anti-CD34 (1:50, Monosan) and rabbit anti-Ki67 (DAKO), followed by incubation steps in biotin-labeled goat anti-mouse (1:400, DAKO), normal mouse serum (1:100, Sanquin), FITC-conjugated mouse anti-CD3 (1:20, DAKO) and a mixture of Alexa 568 labeled streptavidin (1:80, Molecular Probes, Eugene, Oregon, USA) and Alexa 633 labeled goat anti-rabbit antibody (1:800, Molecular Probes) before mounting in Vectashield (Vector Laboratories Burlingame, CA, USA). Between incubation steps, the slides were rinsed extensively in PBS.

*In vivo experiments*

All animal procedures were approved by the University of Arkansas for Medical Sciences Institutional Animal Care and Use Committee (IACUC Protocol #3836). Briefly, 5-weeks old Foxn1^-/-^ female mice were obtained from The Jackson Laboratory (#002019). Human renal tumors were established by transplanting 0.85 mg Caki-1 (HTB-46; ATCC) tissues subcutaneously on the right hind flank. After 12 days, when tumors reached 150 mm^3^ on average, mice were randomized and divided (n=5 per group). Sunitinib (20 mg/kg/day) treatment was administered by oral gavage, whereas the control mice received the vehicle only p.o. (6.25% DMSO). Tumor size was measured and tumor volume was calculated by the equation for volume of a spheroid: (a^2^ x b x π)/6, where a is the short axis and b is the long axis of the tumor. On the last day of the experiment, tumors were measured and resected after humanely euthanizing the animals according to the 2020 AVMA Guidelines on Euthanasia (<https://www.avma.org/resources-tools/avma-policies/avma-guidelines-euthanasia-animals>).

**Supplementary Figures**


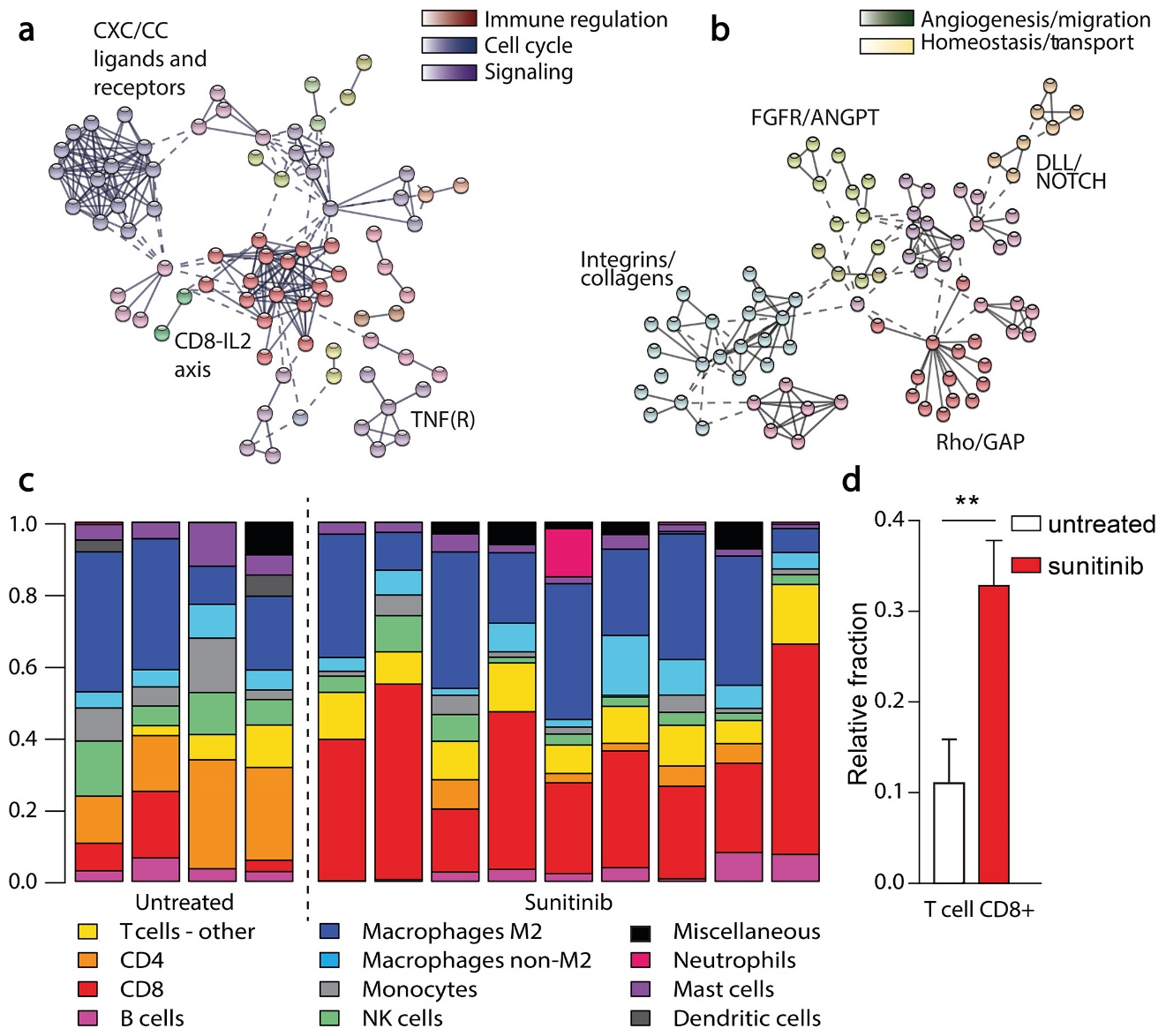


**Fig. S1.** *RNAseq analysis of RCC tumor tissues after pre-surgical treatment with sunitinib.*

**a, b**. Protein-protein interaction analysis of genes relatively upregulated after sunitinib treatment (**a**) or in untreated tumors (**b**). For visual presentation, gene names are omitted and dense clusters of proteins are given the same color. Functional annotation of several clusters is indicated in the text. **c**. RNA based identification of leukocyte subsets using digital flow cytometry with Cibersort. For clarity of visual presentation, several subsets were merged. Data are presented as relative fractions and color coded according to the legend presented below the graph. All patient samples included in the analysis are shown individually. **d**. Average (+/- SEM) of CD8+ cells in untreated vs sunitinib-treated patients as determined by Cibersort, ** p<0.01.


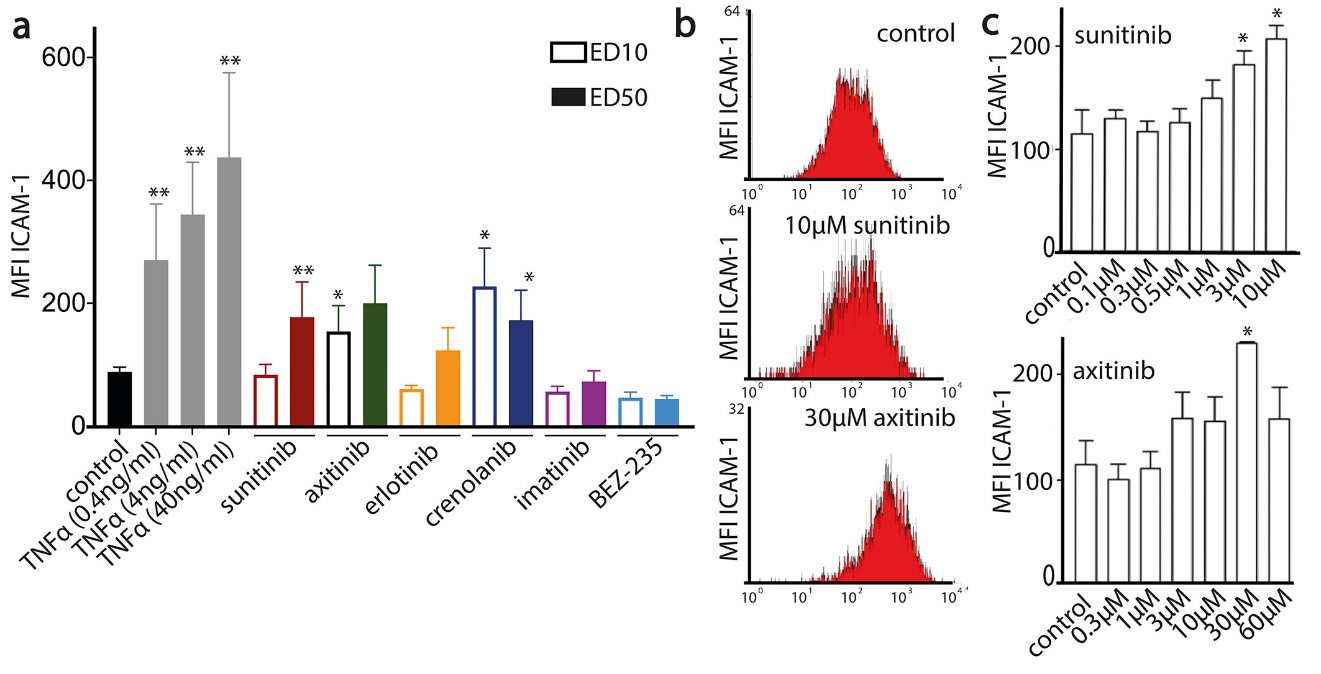


**Fig. S2.** *Induction of endothelial ICAM-1 expression by angiogenesis inhibitors in the ECRF24 human endothelial cell line.*

**a**. Induction of ICAM-1 expression by a selection of angiostatic compounds targeting a variety of signaling pathways in ECRF24 immortalized human endothelial cells. Doses were selected to be the effective dose inhibiting the cell viability for 10% (ED_10_) or 50% (ED_50_). As a positive control exposure to a range of TNFα concentrations was performed (0.4-40 ng/ml). Results are expressed as the mean fluorescence intensity ±SEM. **b** Flow cytometric analysis of surface ICAM-1 expression on ECRF24 cells, exposed for 72 hours to 10 µM sunitinib or 30 µM axitinib. **c**. Dose response plots of ICAM-1 expression induction in ECRF24 by sunitinib and axitinib. Significance in all panels is indicated by * p<0.05, ** p<0.01.


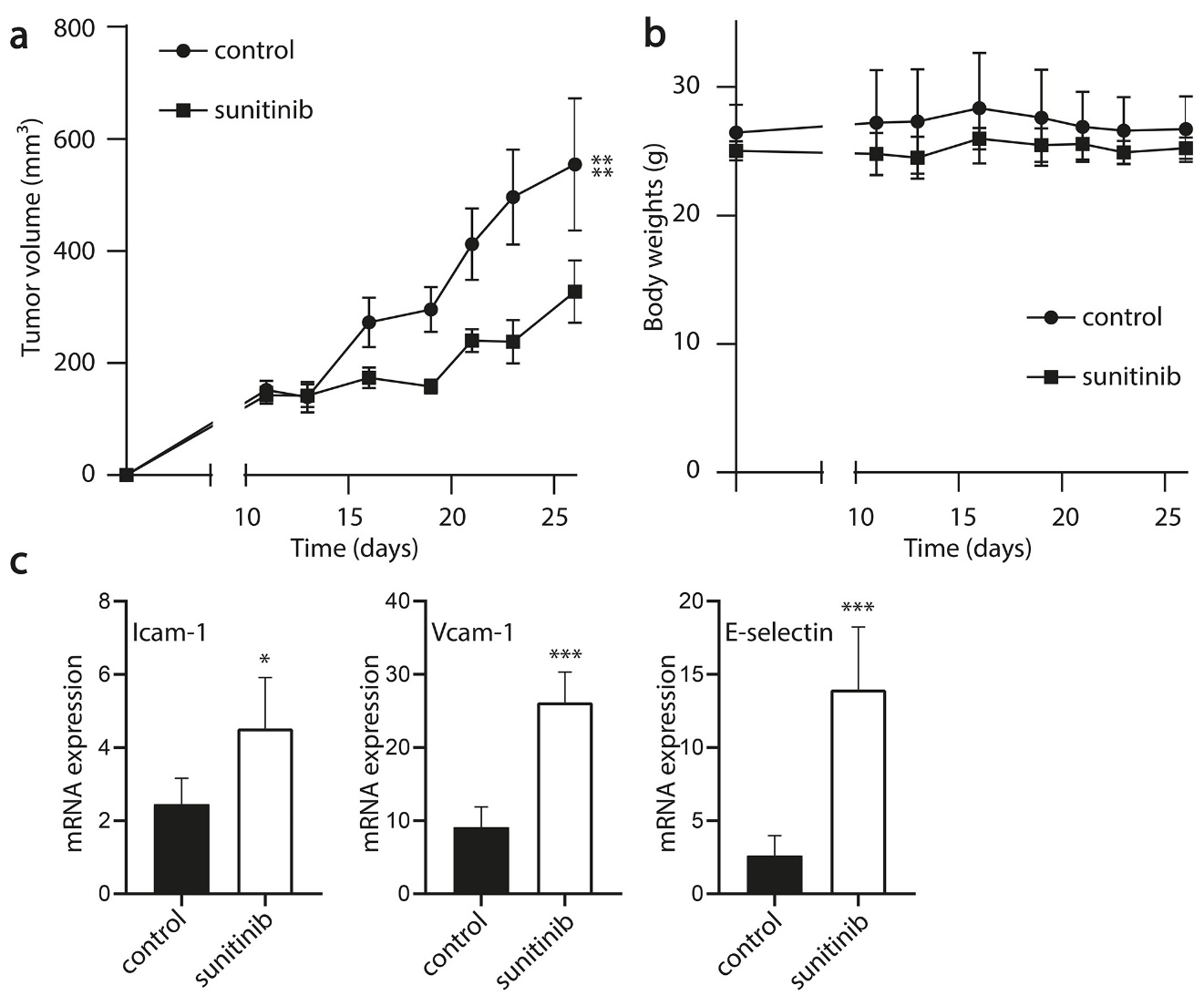


**Fig. S3.** *Induction of adhesion molecules in vivo in a RCC xenograft model.*

**a.** Tumor growth curves of subcutaneous tumors of Caki-1 human RCC cells, treated with sunitinib (20 mg/kg/day) or vehicle (**** p<0.0001), n=5/group. **b**. Body weights of the mice during the study. **c**. qPCR analysis of Icam-1, Vcam-1 and E-selectin expression in tumors of sunitinib vs. vehicle treated mice. Murine-specific PCR primers were used excluding the analysis of tumor cell transcripts. * p<0.05, *** p<0.001.Table S1. Type or paste table title here. Paste table below the title.

Table S1. Species specific quantitative RT-PCR primers used

| **Target gene** | **Forward primer (5'-3')** | **Reverse primer (5'-3')** |
| --- | --- | --- |
| murine Actb^a^ | GAAGCTGTGCTATGTTGCTCTA | GGAGGAAGAGGATGCGGCA |
| murine Ppia^a^ | ATTTCTTTTGACTTGCGGGC | AGCTAGACTTGAAGGGGAATG |
| murine B2m^a^ | CCGCCTCACATTGAAATCC | CTCTGCAGGCGTATGTATCAG |
| murine Cd31 | ATCAGCTGCCAGTCCGAA | AGGTCACCTCGAGAGTCTGG |
| murine Vcam1 | AGTTGGGGATTCGGTTGTTC | CATTCCTTACCACCCCATTG |
| murine Icam1 | GTGGCGGGAAAGTTCCTG | CGTCTGCAGGTCATCTTAGGAG |
| murine Sele | CATGCAAAGCTGTGACCTGTGAC | CCCTGCAACGTGAAACTCTG |
| human ACTB^a^ | TTCCAAATATGAGATGCATT | CCTGTGTGGACTTGGGAGAG |
| human PPIA^a^ | CTCGAATAAGTTTGACTTGTGTTT | CTAGGCATGGGAGGGAACA |
| human B2M^a^ | TCCATCCGACATTGAAGTTG | CGGCAGGCATACTCATCTT |
| human ICAM1 | GGCCGGCCAGCTTATACAC | TAGACACTTGAGCTCGGGCA |
| human VCAM1 | TCAGATTGGAGACTCAGTCATGT | ACTCCTCACCTTCCCGCTC |

^a^Household genes β-actin, peptidylprolyl isomerase A (cyclophilin A) and β2-microglobulin.

**Table S2**: Drugs used and their molecular targets

| **Drug name** | **Molecular Targets** |
| --- | --- |
| Sunitinib / Sutent^®^ | VEGFR2, PDGFR-β, c-KIT |
| Bevacizumab / Avastin^®^ | VEGF |
| Axitinib / Inlyta^®^ | VEGFRs, PDGFR-β, c-KIT |
| Crenolanib / no generic name | PDGFR-α, PDGFR-β, FLT3 |
| Imatinib / Glivec^®^ | BCR-ABL, c-KIT, and PDGFRA |
| Erlotinib / Tarceva^®^ | EGFR |
| BEZ-235 / Dactosilib^®^ | Pl3K, mTOR |
